# Bioconservatism, bioenhancement and backfiring

**DOI:** 10.1080/03057240.2019.1576125

**Published:** 2019-04-01

**Authors:** Tamara Kayali Browne, Steve Clarke

**Affiliations:** a School of Medicine, Faculty of Health, Deakin University, Geelong, Australia; b School of Humanities and Social Sciences, Charles Sturt University, Wagga Wagga, Australia; c Wellcome Centre for Ethics and Humanities, University of Oxford, Oxford, United Kingdom

**Keywords:** backfire, bioconservatism, bioenhancement, Brave New World, conservative, enhancement, moral education

## Abstract

The prospect of enhancing ourselves through the use of new biotechnologies is for the most part, hypothetical. Nevertheless, the question of whether we should undertake such enhancement is worthy of discussion as it may become possible in the future. In this article, we consider one form of argument that conservative opponents of biotechnological means of enhancement (bioconservatives) deploy in opposition to the use of enhancement technologies—the backfiring objection. This is the objection that the use of such technologies is liable to go wrong and lead to outcomes that are inferior to the outcomes intended. We will argue that the objection is not nearly as significant as bioconservatives suppose it to be. Bioconservatives sometimes supplement the backfiring objection by arguing that change will be irreversible, that the new (or the unconventional) is especially liable to backfire and that humans possess severe and permanent limitations which cannot be overcome. We consider these ways of supplementing the backfiring objection and argue that each of them, when properly understood, is of limited value to the bioconservative. We also consider how traditional approaches to moral education can be supplemented by bioenhancement.

## Introduction

The prospect of humans using new biotechnologies to enhance themselves has been the subject of considerable controversy, and has aroused the opposition of ‘bioconservatives’ who are adamant that we ought not to use biotechnologies to enhance ourselves. They raise many distinct arguments against human bioenhancement.^1^ ‘Bioconservative’ is a term that is often used to describe those who wish to conserve humanity as it is, and so oppose human enhancement. Although it is quite possible to be a bioconservative without being a political conservative, many bioconservatives, such as Leon Kass (2003) and Francis Fukuyama (2002), are more generally politically conservative.^2^ It has recently been suggested by a number of recent ‘bioliberal’ and ‘biomoderate’ critics of bioconservatism that, by their own lights, bioconservatives who are also political conservatives should be in favour of at least some enhancements.

In the conservative political tradition, the fixity of human nature is stressed, as is its flawedness (Kekes, 1998, pp. 41–42). Humans, say conservatives, are selfish, impulsive and often motivated by base feelings of envy, pride, spite and so on and moral education will not do anything to significantly alter their characteristics. They also have distinctly limited cognitive capacities and regularly make bad decisions. But, say these recent critics of bioconservatism, the use of human bioenhancement technologies offers us opportunities to overcome the fixity of human nature and ameliorate our flaws. One example of this line of argument being developed is due to Roache and Savulescu (2016). They engage with Fukuyama’s (2002) concern that if we become enhanced post-humans then we may lose some intrinsically valuable feature of being human, which he is unable to define and which he dubs ‘Factor X’. Factor X might include some combination of humility, an appreciation of ‘giftedness’, dignity, ‘openness to the unbidden’, or whatever other feature of humanness may be threatened by bioenhancement (Roache and Savulescu, 2016, pp. 150–151). Their response is to point out that Fukuyama cannot be offering a general objection to human bioenhancement. If Factor X is the intrinsically valuable feature of humanity that ought not to be lost then Fukuyama can have no well-founded objection to enhancing Factor X itself (Roache and Savulescu, 2016, p. 151).^3^ Similarly, it is argued by Kaebnick (2016) that it is difficult for anyone to be straightforwardly opposed to the idea of moral bioenhancement, even for those who are generally opposed to human bioenhancement, as almost everyone is in favour of more moral behaviour.^4^ Because conservatives stress the moral shortcomings of ordinary humans, and see these failures as a barrier to attempts to reform human societies, it seems that they should be particularly in favour of moral bioenhancement. Suitably morally bioenhanced individuals would be more likely, it seems, to help create more cohesive societies—a key conservative ideal—than the many un-morally enhanced, vice-ridden individuals who we currently find beside us in unenhanced human societies.

A way conservatives can respond to the above lines of reasoning is to concede that a society in which Factor X and/or morality was suitably enhanced would be preferable to current societies but to argue that we should not attempt to create such a society because attempts to do so are liable to backfire and lead, inadvertently, to the creation of societies that are worse, rather than better, than current human societies. Appeals to ‘backfiring’ have long been a key weapon in the conservative intellectual arsenal; and conservatives have long been opposed to social engineering projects on the grounds that these are likely to backfire (O’Hara, 2011, pp. 53–57). Some of the most prominent conservative objections to human bioenhancement are backfiring objections. Conservatives are concerned that well-meaning schemes to improve society by the use of human bioenhancements on a mass scale will backfire and lead to some or other outcome that is worse than what we have now.^5^

A possible response to the conservative appeal to backfiring would be to argue that even if backfiring is likely to occur it is worth taking the risk of it occurring for a chance at enhancing ourselves successfully. The conservative has several ways of replying to this response. The first is to stress that backfiring is very likely to occur. The second is to suggest that the benefits that could be obtained by successful schemes to improve ourselves are not as great as their proponents imagine them to be. We might, for example, succeed in making ourselves smarter than we are now, but there is a lack of evidence to suggest that this would make us happier than we are now (Clarke 2016b, p. 384). Similar lines of objection can be run against other proposed enhancements. A third way to respond is to argue that we ought to adopt a particular attitude to risk, including the risk of backfiring: we should be risk averse. Almost all conservative writers advocate an attitude of risk aversion.^6^ Conservatives do not usually use this terminology, but their attitude to risk is very similar to the attitude of advocates of the influential ‘precautionary principle’.^7^ It would take us too far afield to respond to this conservative reply in detail here, but it is worth pointing out that conservative authors do not have much to say in favour of a risk averse attitude other than to point out that it seems common-sensical. The precautionary principle is very popular but it suffers from a number of well-known conceptual problems, and so it is far from obvious that adopting the precautionary principle would help to buttress the conservative case for a risk-averse attitude.^8^

We will argue that the backfiring objection to human bioenhancement is not nearly as significant as conservatives often suppose it to be. Conservative opponents of human enhancement sometimes supplement the backfiring objection by arguing that change will be irreversible, by arguing that the new (or the unconventional) is especially liable to backfire, and by arguing that humans possess severe and permanent limitations which cannot be overcome. We consider these ways of supplementing the backfiring objection and argue that each of them, when properly understood, is of limited value to the conservative.

For the purposes of this article we will adopt a constructivist approach to the definition of ‘enhancement’. In contrast with other approaches to the definition of ‘enhancement’, such as the welfarist approach, the normal functioning approach and the beyond-species-typical approach, the constructivist approach emphasises the role of societal norms and values in what is considered a disease (and conversely, what is considered an enhancement). According to Gyngell and Selgelid, ‘Under [the Constructivist Approach] enhancements are individually beneficial alterations to human functions or capacities which are not performed in the context of treating or preventing disease, with diseases being understood as states which are disvalued by society in a particular way’ (2016, p. 112).

### Brave New World

A prominent theme in bioconservative literature is the propensity of attempts to enhance humans to lead to the inadvertent creation of a dystopic society, such as the one depicted in Aldous Huxley’s *Brave New World* (1932/2004).^9^ This appears to be a concern about potential backfiring. The society depicted in *Brave New World* (1932/2004) is one in which a world government breeds humans to be members of distinct castes—Alphas, Betas, Gammas, Deltas and Epsilons—that play designated roles in the world economy. The castes are differentiated by their levels of intelligence, however, no one is cognitively enhanced in the society depicted in *Brave New World*. Gammas, Deltas and Epsilons are cognitively dis-enhanced by being doused with alcohol and deprived of oxygen while they are foetuses. Alphas and Betas are bred to have the intelligence necessary to function effectively in their designated economic roles. There is no manipulation of the genetic material of members of these castes to produce higher-than-natural levels of intelligence and no mention of any other procedure that might plausibly be counted as a form of cognitive enhancement. Huxley does not explain how he imagines that the society of his time might turn into the one depicted in *Brave New World*.^10^ But if it was a result of an attempt to create enhanced humans (as both Fukuyama (2002) and Kass (2003) appear to suppose) then it is one that backfired comprehensively.

The society depicted in *Brave New World* is usually taken to be dystopic. But as Fukuyama concedes, it is not immediately apparent what is wrong with that society. As he points out, ‘no one is hurt’ and ‘everyone gets what they want’ (2002, p. 5). One problem with the society depicted in *Brave New World* that Fukuyama points to, is that the residents of it are not the sort of beings we would like to become. As he points out: ‘They no longer struggle, aspire, love, feel pain, make difficult moral choices, have families, or do any of the things that we traditionally associate with being human. They no longer have the characteristics that give us human dignity’ (Fukuyama, 2002, p. 6). In a somewhat similar vein, Kass suggests that the central problem raised by *Brave New World* is the problem of ‘dehumanization’ (2003, pp. 15–16). We feel a rising sense of unease when reading Huxley’s depiction of this society because the apparently human creatures within it reveal themselves to behave so differently from us that we start to doubt that they share our ‘human nature’ (Fukuyama, 2002, p. 7). It appears that they have become post-human (Kass, 2003, p. 10), and so, should be considered a different species from us.

We could imagine someone digging their heels in and disputing the widespread assumption that the society depicted in *Brave New World* actually is a dystopia. They might point out that the lives of the vast majority of the residents of that society are happy and secure, and point out that the vast majority of them show no desire to change those lives. So, what if they are post-human? Wouldn’t it be better for us to become happy, secure post-humans than remain miserable, insecure humans? If the use of human bioenhancement technologies led us to inadvertently become a society like the one depicted in *Brave New World* then that is a fortuitous outcome for us, rather than a case of backfiring, or so a defender of the society depicted in *Brave New World* might argue.

Fukuyama concedes that it is difficult to pinpoint exactly what is wrong with the society depicted in *Brave New World* (e.g., Fukuyama, 2002, p. 5). But without being able to articulate a clear objection to the society depicted in *Brave New World* he would stand very little chance of persuading a defender of that society to change their mind. However, neither he nor Kass are aiming to convince such a person. They are relying on the widespread intuition that there is something wrong with the society depicted in *Brave New World*, to persuade us to accept that we should also be motivated to accept that mass-scale attempts to bioenhance humans ought to be avoided, on the grounds that they are liable to backfire and lead to a *Brave New World*-like scenario.

While Fukuyama (2002) and Kass (2003) worry about mass-scale schemes to bioenhance humans backfiring, other authors are concerned with the possibility of individual bioenhancements backfiring. Agar (2013) considers the widely canvassed possibility of artificially enhancing an individual’s sense of empathy, in order to make them more moral. Both individual and societal level attempts at bioenhancement might backfire and the bioconservative is entitled to object to both.^11^ Agar argues that moral judgement consists of a balance of emotional, motivational and cognitive factors and that the bioenhancement of any of these factors could well throw moral judgement off-kilter. He presents two widely-held moral ideas:

Moral idea one: Beings with a similar capacity to benefit and suffer harm deserve similar treatment.

Moral idea two: Parents should give special consideration to their children. (Agar, 2013, p. 344)

Agar argues that moral bioenhancement need not lead one to let go of either of these ideas for a misguided trade-off to result. He contemplates a specific instance of backfiring in which an attempt to enhance a mother’s empathy, in order to improve her moral judgement, leads to a situation where she ‘breaks into a hospital, steals a dialysis machine, and sells it on eBay to provide her child with a higher quality of education’ (Agar, 2013, p. 344). In this case the mother’s overriding sense of empathy for her child has led her to tip the balance between moral idea one and moral idea two in favour of the latter. So, an attempt to enhance moral judgement has backfired.

## Fear of unconventional methods

Conservatives such as Kass and Fukuyama raise concerns about the potential for biotechnological human enhancements to backfire, but do not appear to be concerned about the potential for ‘conventional’ human enhancements to backfire. We are familiar with conventional means of attempting to improve oneself. These include personal introspection, moral education, performing good deeds, making a concerted effort to behave well, meditation and religiosity. While the first five seem quite low-risk, the last one has the potential to be dangerous. Not only do religions tend to have their own, specific (and sometimes eccentric) views about what it is to be a good person (Boyer, 2001, pp. 192–198), but following religious instruction in order to improve oneself can sometimes be understood as involving judgement, ostracism and even punishment of oneself and others. While religion can be practised in liberal and tolerant ways, it can also be practised in intolerant and fundamentalist ways. Many conservatives are also religious fundamentalists. There are numerous instances throughout history of religious wars and persecution (e.g., the Crusades, the Thirty Years’ War and the Spanish Inquisition) where it seems that attempts to become better (by attaining or keeping to a certain faith, or at least one’s interpretation of it) have backfired and led to participation in gratuitous violence.^12^ Furthermore, religiosity, while only one means by which people attempt to improve themselves, is one of the most popular means, not just in the present day, but throughout history. If anything could be considered the *status quo* when it comes to attempts at self-improvement, religiosity would certainly be one of them. Yet it is not clear that biotechnological means of human enhancement would be any more likely to backfire than religious means. Until significantly many biotechnological means for human enhancement have been developed, it is difficult to speculate as to their degree of risk of backfiring.

If we were to examine what it is about religion as a means of self-improvement that makes it liable to backfire so dramatically, the following are possible contenders:

1. Many followers of fundamentalist religions adhere to divine command theories of morality. While the apparently divine origin of these moral prescriptions does not preclude them from critique, it makes such critique more difficult compared with those of other philosophies and ideologies that do not purport to originate from non-human sources.
2. Humans are required to interpret and enact divine commands. Unlike the deities which issue divine commands, humans are flawed and can misinterpret divine commands. However, when they do so they often remain convinced that their interpretations of divine commands are precluded from critique. (Unlike the first point, this point does not dispute the divine origins or authenticity of religious messages but the way these are interpreted.)^13^

In contrast, biotechnological means of human enhancement do not appear to have the above risks associated with them. The rationale behind enhancement of one’s self is something one can question and argue for or against, as opposed to a divine commandment that is usually treated as infallible. Regarding the second contender, biotechnological means of human enhancement have the distinct advantage of being able to bypass many human character defects, since the only human decision involved is whether or not to take the enhancement in the first place. In fact, it may be that if enhancement can cause certain character flaws (such as selfishness, greed, a fear of the unfamiliar or an aversion to certain racial groups) to disappear, religious attempts at self-improvement may be less likely to backfire. In other words, bioenhancement could cause humans to be less flawed and therefore less likely to have flawed interpretations of the divine commands. A combination of techniques may even mitigate the risk of either method backfiring. The overriding point here is that it does not make sense to worry that biotechnological enhancement will backfire without also worrying that ‘conventional’ methods will do the same. ‘Conventional’ means can, and do, backfire due to factors that will not be present in biotechnological enhancement. Further, biotechnological enhancement could even reduce the risk of backfiring associated with conventional methods of self-improvement such as religion.

## Irreversibility

This brings us to another point that is often overlooked by bioconservatives who appeal to concerns about backfiring in debates about bioenhancement—the possibility that even if an enhancement does backfire, the effects of backfiring can be reversed. There are, of course, examples of interventions whose damage has been permanent or hard to reverse, such as the effect of the industrial revolution on global warming (e.g., Abram et al., 2016), or the introduction of non-native animals, such as rabbits and cane toads, that have become pests in Australia (NSW Office of Environment and Heritage, 2015a, 2015b). Yet there are also interventions whose negative consequences, when first introduced, were successfully overcome. For instance, when electricity was first introduced into households, many deaths were caused by uninsulated wires and uncertainty as to how to use it (e.g., attempting to light the socket with a match) (BBC, 2013). This problem diminished in significance very quickly as people became more familiar with electricity.

There is a conventional method by which unconventional interventions can be tested and improved before being made widely available—controlled trials. There is no reason why the pursuit of biological or technological means of human enhancement should not follow the same procedures currently followed by clinical trials such that, even when it first reaches the human trial stage, only a limited number of individuals would initially be tested so that any ‘backfiring’ effects that might occur are controlled. By trialling potential human enhancements before making them available, we can learn how particular human enhancements are liable to backfire. This can help us to limit the likelihood that backfiring will occur in the first place. It can also help us to learn how to reverse instances of backfiring that do occur.

Of course, there are limits to the capacity of controlled trials to enable us to minimize and then reverse the effects of backfiring. We may be unable to find ways of reversing some forms of backfiring. Also, it may be that some forms of backfiring only occur when enhancements are introduced at a mass scale. We can conduct controlled trials on individuals and on small groups easily enough, but it is often going to be impractical, or unacceptably harmful, to conduct controlled trials on large groups, so we will not usually be able to determine how to restrict or reverse backfiring effects that only arise at the mass scale.^14^

## The inevitability of backfiring

Why are many conservatives convinced that social engineering almost invariably leads to backfiring? One answer they might give is an inductive one. On many past instances it has been observed that attempts to socially engineer society have backfired and led to suboptimal outcomes (O’Hara, 2011, pp. 52–55). Conservatives often provide examples to illustrate this tendency. For example, it is frequently noted by conservatives that the various planned economies of the twentieth century communist bloc were economic and political failures.^15^ Also, genuine conservatives, such as Fukuyama (2006, pp. 29–31) will cite the failures of George W. Bush’s neo-conservative administration to re-engineer Iraq and Afghanistan as liberal democratic societies as confirmation of the wisdom of traditional conservative strictures against human enhancement. However, there is a danger here of cherry picking the evidence. It might seem that conservatives ignore successful instances of social engineering. The 1933–38 social and economic initiatives that together constituted Roosevelt’s ‘New Deal’ are often regarded as comprising a successful instance of social engineering. Similarly, the ‘Marshall Plan’, instituted to rebuild war-torn Europe after WWII, involving the liberalisation of trade within Europe, backed up by a massive injection of American money, looks like a successful instance of social engineering. Conservatives can attempt to respond to these examples by arguing that they involved less extreme departures from the political and economic status quo than more radical instances of social engineering which they find objectionable, such as the attempt to turn traditional societies into communist societies^16^ and attempts to enhance significant numbers of humans. Also, there are instances of radical social and biological engineering that, while imperfect, are considered to have been largely successful. For example, the American revolution, the widespread use of contraception, the abolition of slavery, the US civil rights movement and the introduction of women’s suffrage.

One may well ask whether/why it is that conservatives believe that newer forms of social or biological enhancement should be more likely to backfire than older forms. An intervention is not doomed to backfire simply because it is new. (Interventions here being a wider category but including enhancements.) Interventions both new and old have been known to backfire. Torture for the purpose of extracting information, for instance, is an old intervention which is known to backfire frequently, resulting in false confessions and mis-information (Costanzo, Gerrity, & Lykes, 2007; Rejali, 2009). However, relatively new interventions such as the use of antiretroviral drugs are considered safe and effective in the treatment of HIV/AIDS with known, manageable side-effects (World Health Organization, 2016). There are, of course, also examples of new interventions which have backfired. That an intervention is new or old has no bearing on its potential to succeed or backfire. In any case, there are many social/technological/biological interventions, which were new interventions at some stage, since much has changed since hunter-gatherer days. Were humans wrong to pursue enhancements to ourselves and our way of life, since they were all, at some stage, new and drastic? If conservatives answer no to this question, then they need to explain why new and drastic enhancements were permissible at an earlier time but are no longer permissible—why they are now particularly likely to backfire, when they were not as likely to do so before. If they cannot do this then they are vulnerable to the charge that they are merely opposed to new enhancements because they suffer from status quo bias.^17^

## Human nature’s constraints

Another approach to defending the conservative backfire view is to ground it in considerations of human nature. A key tenet of political conservatism is the view that human nature imposes severe constraints on the very possibility of deliberately improving human society (Kekes, 1998, p. 41).^18^ Humans suffer from severe cognitive and affective limitations and so are not capable of deliberately improving the complicated societies they inhabit, or so conservatives argue. We have two objections to this contention. First, if conservatives really believe this, one might wonder whether they believe there is any value in education. If our cognitive and affective limitations are so severe that we cannot improve our societies, there would appear to be little point in attempting to do so either by traditional means (such as education) or novel means (such as bioenhancement). We do not agree with this contention and believe there is not only a point in both education and bioenhancement, but that the two could, and should, work together to improve humans.

The arrival of a new technology is often accompanied by the need for education to enable people to use the technology effectively. In the same way, bioenhancement would likely need to be accompanied by education in order to enable people to understand its purpose, who should use it and how and when it should be used. In fact, if bioenhancement is to be used to improve society, then ideally some guidelines around its use should be developed and distributed to the public.^19^ Doing so would maximise the chances that it will be used responsibly and by those who need and will benefit from it, and minimise the risk of it backfiring. Even if a government decides to control access to bioenhancements rather than having them available on the consumer market, people would still need to be educated as to why that is the case. Likewise, bioenhancement could also be beneficial to education if there are bioenhancements that enable people to become more receptive to education, or enhance their ability to make the most of their education, moral and otherwise. Perhaps bioenhancement could even motivate people to seek out more education. Either way, bioenhancement and education could work together to improve society in a way that may well be more than the sum of education and bioenhancement used separately.

Second, if conservatives are right about human limitations, we might wonder how we can continue to maintain and develop the complicated societies we currently have, given these cognitive and affective limitations. The answer, according to mainstream conservatives, is that we rely to a large extent on a large stock of implicit, non-conscious knowledge. We have built up social institutions that work for us, by trial and error, over the centuries.^20^ However, we cannot rely on this stock of implicit knowledge when attempting social engineering. Instead we must rely only on our meagre stock of conscious explicit knowledge. But this is not sufficient to enable us to reliably re-engineer our own societies. There are a number of problems with this objection. Conservatives need to provide evidence for the claims:

1. that we rely significantly on ‘implicit knowledge’, rather than ‘explicit knowledge’ when attempting social engineering;
2. that we cannot rely on ‘implicit knowledge’ to reliably re-engineer our societies;
3. that our stock of ‘explicit knowledge’ is meagre; and
4. that our stock of ‘explicit knowledge’ is not sufficient to reliably re-engineer our societies.

Allen Buchanan (2011) has considered the conservative backfiring objection against social engineering in some detail. On his view the conservative who relies on this objection places herself in an ‘uncomfortable position’. She must show that humans have sufficient cognitive power to know that they have severe and permanent cognitive limitations and to appreciate that traditional social arrangements embody superior knowledge, but insufficient cognitive power to overcome these limitations (p. 150). Suppose that a conservative did advance an argument for the conclusion that our cognitive powers were in the conservative ‘sweet spot’—powerful enough to appreciate our cognitive limits but not powerful enough to allow us to overcome these limits. The conservative would then be faced with a second objection. Advances in the social sciences threaten to make social engineering more reliable than it has been previously by increasing our stock of explicit knowledge and reducing our reliance on implicit knowledge, and so they threaten to move us out of the conservative sweet spot (p. 151). To head off this objection the conservative needs to demonstrate that the social sciences could never advance to such an extent that we would acquire sufficient explicit knowledge of our own society to enable reliable social engineering. In other words, they need to show that there are hard limits to the ability of social scientists to find out how societies function.

The place to look for reasons to think that there are hard limits to the ability of social scientists to know how societies function is in the philosophy of the social sciences. Conservatives need to deny ‘naturalism’ in the philosophy of the social sciences: the view that the social sciences are continuous with the natural sciences and are capable of furnishing us with explanations that have the potential to be as reliable as those furnished by any other science.^21^ The sort of views in the philosophy of the social sciences that should appeal to conservatives—because they provide support for the view that the social sciences will never provide the knowledge needed to underpin social engineering projects—are views that assert that there is a sharp divide between social and natural science. One set of views that should be helpful to conservatives are hermeneuticist views which have it that the social sciences are not, and cannot be, directed at explanation and that, at best, the social sciences can only provide us with an understanding of our social circumstances, including, perhaps, understanding that the social world cannot be satisfactorily explained and controlled to the extent needed to underwrite social engineering projects.^22^ Similar support can be found from Wittgensteinian scholars, such as Winch (1958), who argue that the social sciences are not and cannot be aimed at uncovering the causes of human behaviour, but have a different goal instead, which is to clarify the reasons for human action. If they are right, then the causal consequences of social engineering projects are doomed to be forever mysterious to us. It would take us too far afield to go over the pros and cons of the various alternatives to naturalism that are available in the philosophy of the social sciences.^23^ Suffice to say that the debate is far from settled.

If the conservative is able to make the case for the conclusion that the social sciences cannot provide sufficient social knowledge to underpin social engineering projects then they have done half of what is needed to satisfy Buchanan, but they still need to do more. They also need to provide us with good reason to suppose that we have sufficient knowledge to know that we have severe and permanent cognitive and affective limitations which cannot be overcome with the use of bioenhancement technologies. It might well be that cognitively enhanced individuals will be able to consciously comprehend much more about our current society than we are currently capable of comprehending (Buchanan, 2011, p. 151). And it seems plausible to think that we can cognitively bioenhance a few individuals without making significant alterations to our social arrangements.

What appears to be getting bioconservatives into intellectual trouble is adherence to the traditional political conservative view that humans have severe and permanent limitations that cannot be overcome. As Buchanan points out, the requirement that they substantiate this view makes it very difficult for them to also make a convincing case against social engineering. Also, it opens the door for opponents of bioconservatism, including Roache and Savulescu (2016) and Kaebnick (2016) to argue that they ought to be in favour of bioenhancements that enable those severe and permanent limitations to be overcome.

What becomes of their opposition to bioenhancement if bioconservatives abandon dogmatic views about the severe and permanent constraints of human nature? They can run the same objections, but the objections themselves become pragmatic ones. The bioconservative can no longer claim to be able to show that attempts to bioenhance humans will invariably backfire; and must settle for the conclusion that attempts to bioenhance humans may well backfire. This is still a worthwhile point to make, but it is not strong enough to underwrite an absolute prohibition on attempts to bioenhance humans.^24^ At most it can substantiate the claim that we should be very cautious about any and every attempt to enhance humans, because like other social engineering projects these have a tendency to backfire. An additional pragmatic point that the bioconservative can make is that the relationship between human biology and human social organisation is complicated and can be delicate. To the extent that schemes to enhance large numbers of humans interfere with this relationship they may cause unanticipated harms or backfire disastrously.^25^ But again, this is a pragmatic point, which should lead us to approach human enhancements cautiously, rather than reject them outright.^26^

## Bioenhancement and moral education

Earlier on, we rejected conservative pessimism about the possibility of effective moral education based on the view that human nature imposes severe constraints on the possibility of altering human character traits, including moral virtues. We also suggested that bioenhancement techniques could be combined with more traditional approaches to moral education to enable moral enhancement.^27^ In this section, we expand on these remarks. The emerging biotechnologies that may enable bioenhancement are not the only technologies that could be used in the future to enable moral enhancement. Artificial intelligence also has this potential.^28^ However, our focus is on the potential for integrating bioenhancement technologies with more traditional approaches to moral education to make moral education more effective than it is currently.

The most promising way of integrating moral education with bioenhancement would be to use new biotechnologies to speed up the learning experience, thereby enabling more to be learned in a given time period. One way of doing this would be to use new biotechnologies to heighten attention and reduce fatigue, enabling a more sustained learning process.^29^ If a bioenhancement were to become available that enabled one to learn more quickly, it could make a parent or educator’s role of instilling moral virtues and other good habits easier and more effective.

A second approach would be to use memory enhancing biotechnologies to enable students to better recall moral lessons.^30^ While much moral education consists in helping students to ‘cultivate the virtues’ students also need to learn and recall facts. This is particularly the case when students are taught by appeal to narratives describing exemplars of morally appropriate behaviour. Students need to have the capacity to recall key features of such narratives in order to apply lessons contained in them to real world situations; and this capacity could be enhanced. A third approach would be to integrate new biotechnologies with current moral educational techniques to help children and adults to learn to modulate their emotions. For instance, anger management classes are offered to those who are convinced of the moral value of curtailing their anger but who may lack the skills to do so. Learning to change one’s behaviour after years or decades of problematic habitual responses can be difficult in practice, even for those who have every intention of improving themselves (Walker & Bright, 2009). Moral bioenhancement may help students to enact the skills one learns at anger management classes (e.g., by helping them to overcome certain instinctive emotional or behavioural responses, and to modulate their emotions).^31^

Here we have considered three ways in which traditional approaches to moral education could be supplemented by bioenhancement technologies. We do not wish to rule out the possibility that traditional approaches to moral education could be replaced by the use of bioenhancement technologies. We see this as a far off possibility, however. Nowadays, we are accustomed to hearing stories of people who gained a moral education by experiencing life-changing events, overcoming hardship or trauma, travelling, serving others and so on, prompting them to gain significant insights, transform their lives and become better people. Consider, for example, the veteran who returns from war and becomes a peace activist (Aderet, 2018; Mehren, 2006), or the prisoner who becomes a role-model for others to turn away from crime (O’Sullivan, 2001). If we could gain the equivalent effects on our persona and behaviour via bioenhancement alone rather than by life experience, then those against it would need to articulate why we should not. In other words, do means matter? As far as we can see, in this context, the answer is no.

## Conclusion

In our view bioconservatives should abandon wholesale opposition to human bioenhancement based on the backfiring objection. This does not mean that they should abandon all opposition to particular bioenhancements. Bioconservatives can and should oppose particular bioenhancements when they can establish that the risk of these backfiring, and causing harm to us, outweighs the benefits that the introduction of such enhancements can reasonably be expected to provide. Such opposition, grounded in cost-benefit analysis, is not peculiarly conservative. It is a pragmatic form of opposition which can also be embraced by non-conservatives.

## Notes

1. For a recent survey, see Giubilini and Sanyal (2016).

2. A clear example of a bioconservative who is not a political conservative is Rob Sparrow. For further discussion of the relationship between political conservatism and bioconservatism, as well as between political liberalism and bioliberalism, see Parens (2005).

3. Roache and Savulescu go on to consider ways in which bioconservatives might try to ground opposition to Factor X enhancement (2016, pp. 151–157).

4. Kaebnick considers reasons why one might be opposed to particular ways in which moral enhancement might be carried out. One might be concerned, for example, that the implementation of particular schemes to enhance humans might undermine freedom and might ‘open the door’ to other objectionable forms of enhancement (2016, pp. 228–234).

5. Not all conservative objections to human enhancement are backfiring objections. Conservatives are also concerned about the unforeseen or under-appreciated side effects of successful enhancement. For example, Sandel (2004) worries that if we were successfully enhanced we would no longer be ‘open to the unbidden’ to the extent that we are now. This appears to be a concern about an under-appreciated side effect of successful enhancement.

6. See, for example, O’Hara (2011, pp. 86–93), Beckstein (2015) and Brennan and Hamlin (2004, pp. 676–682).

7. For exploration of the commonalities between conservative attitudes to risk and the attitudes of advocates of the precautionary principle, see Briggle (2014) and Turner (2010).

8. For discussion of these conceptual problems, see Sunstein (2005) and Clarke (2009).

9. See, for example, Fukuyama (2002, pp. 3–10) and Kass (2003, p. 11).

10. He does offer some thoughts on how this might have occurred in Huxley (1958/2007).

11. It is far from obvious whether we should be focusing on individual or societal programs of enhancements. Whether one or another focus turns out to be more important will depend on how specific attempts to enhance humans play out.

12. For further discussion, see Avalos (2005) and Juergensmeyer (2003).

13. For an extended discussion of divine command theories of the relationship between morality and religion, as well as ‘natural law theories’, see Gensler (2016).

14. It is possible and practical to trial some social reforms on a large scale without causing significant harm. For example, Finland recently completed a two-year trial of the effects of providing a universal basic income to its citizens. A monthly income of 560 Euros was provided to 2000 Finns selected at random. The trial aimed to discover effects on individuals, as well as mass-level effects on the social security system in Finland. For further information about the trial see: https://www.kela.fi/web/en/basic-income-experiment-2017-2018 (accessed 19 February 2019).

15. For example, Scruton (2014, p. 9).

16. Although it is difficult to disentangle whether it is communism that failed or simply dictatorships, since so far the transformation to communism has not involved a ‘dictatorship of the proletariat’ as Marx and Engels envisioned (apart from the Paris Commune, which only survived for two months).

17. For further discussion of the appropriate use of the charge of ‘*status quo* bias’ in the context of enhancement debates, see Clarke (2016b).

18. For further discussion of the importance of appeals to an invariant human nature in the conservative tradition, see Kekes (1998, pp. 49–54).

19. Such education could take the form of information leaflets included as a package insert with the bioenhancement, pamphlets distributed at medical clinics or chemists, or even media advertisements.

20. For further discussion, see Kekes (1998, pp. 48–67).

21. For further discussion of naturalism in the social sciences, see Rosenberg (2015, pp. 11–34).

22. See Geuss (1981) for a clear exposition of hermeneuticist ideas.

23. For recent surveys see Rosenberg (2015) and Bishop (2007).

24. Buchanan argues similarly (2011, p. 151).

25. This argument is developed at greater length in Clarke (2016a).

26. Although the backfiring objection is a line of argument that is pursued most prominently by conservatives, there is nothing distinctively conservative about it. Non-conservatives who favour the pursuit of human enhancement projects should be as concerned about the potential for these projects to backfire as are conservatives. It is not just conservatives who wish to avoid living in a *Brave New World*. Pretty well all of us wish to avoid this outcome.

27. For examples of the more conventional approaches to moral education that we have in mind, see Hand (2017) and Vozzola (2014).

28. For further discussion, see Giubilini and Savulescu (2018).

29. For recent discussion of the use of attention-enhancing drugs, see Greely et al. (2008).

30. For discussion of our prospects for enhancing memory as well as some ethical issues raised by the enhancement of memory, see Liao and Sandberg (2008).

31. For discussion of the prospect for using emotion modulating biotechnologies to improve moral deliberation, see Douglas (2013).
